# Functional contributions of glutamate transporters at the parallel fibre to Purkinje neuron synapse–relevance for the progression of cerebellar ataxia

**DOI:** 10.1186/2053-8871-1-3

**Published:** 2014-06-16

**Authors:** Emmet M Power, Ruth M Empson

**Affiliations:** Department of Physiology, Brain Health Research Centre, University of Otago School of Medical Sciences, PO Box 56, 9054 Dunedin, New Zealand

**Keywords:** Cerebellum, Glutamate transporters, Parallel fibres, Plasticity, Facilitation, Ataxia

## Abstract

**Background:**

Rapid uptake of glutamate by neuronal and glial glutamate transporters (EAATs, a family of excitatory amino acid transporters) is critical for shaping synaptic responses and for preventing excitotoxicity. Two of these transporters, EAAT4 in Purkinje neurons (PN) and EAAT1 in Bergmann glia are both enriched within the cerebellum and altered in a variety of human ataxias.

**Results:**

PN excitatory synaptic responses and firing behaviour following high frequency parallel fibre (PF) activity commonly encountered during sensory stimulation *in vivo* were adversely influenced by acute inhibition of glutamate transporters. In the presence of a non-transportable blocker of glutamate transporters we observed very large amplitude and duration excitatory postsynaptic currents accompanied by excessive firing of the PNs. A combination of AMPA and mGluR1, but not NMDA, type glutamate receptor activation powered the hyper-excitable PN state. The enhanced PN excitability also recruited a presynaptic mGluR4 dependent mechanism that modified short term plasticity at the PF synapse.

**Conclusions:**

Our findings indicate that reduced glutamate transporter activity, as occurs in the early stages of some forms of human cerebellar ataxias, excessively excites PNs and disrupts the timing of their output. Our findings raise the possibility that sustaining cerebellar glutamate uptake may provide a therapeutic approach to prevent this disruption and the glutamate excitotoxicity-induced PN death that signals the end point of the disease.

## Background

The timing of excitatory synaptic responses at mammalian glutamatergic synapses relies upon the rapid recruitment of postsynaptic glutamate receptors and the rate of clearance of glutamate from the synaptic cleft. Glutamate clearance relies upon glutamate transporter carrier proteins (excitatory amino acid transporters, EAATs) located in close proximity to the synapse [1] that couple glutamate movement with the Na^+^ electrochemical gradient, H^+^ movement and a countertransported K^+^ ion [2]. Furthermore, these transporters also behave like ligand gated ion channels, since glutamate activates their inbuilt chloride conductance [3]. The functional importance of the glutamate transporters in the cerebellum is highlighted by the appearance of mutations in cerebellar-enriched EAAT1 in human episodic ataxia type 6 [4–6], the down-regulation of EAATs in animal models of the human spinocerebellar ataxias SCA1, 5 and 7 [7–10] and the mildly ataxic phenotype of mice lacking EAAT1 [11]. Interestingly, in episodic ataxia type 6, a mutation in EAAT1 enhances this transporter’s anion conductance [4] suggesting a potential role for these anion channels in disease.

The main output neurons of the cerebellar cortex, the Purkinje neurons (PN), are highly enriched for EAAT4 [12] present exclusively within the PN somatodendritic compartment [12] and perisynaptic regions [13]. This location means it powerfully influences both climbing fibre (CF) and parallel fibre (PF) synapse responses [14]. Furthermore, since EAAT4 also exhibits the largest glutamate activated chloride (anion) conductance it also has the potential to directly modify the excitability of the PN dendrite [15]. EAAT4 expression is heterogeneous across the cerebellar cortex and enhanced levels coincide with the expression of zebrin II-positive stripes [13] and elevated glutamate release from CF terminals [16]. Under certain conditions the elevated EAAT4 in zebrin II-positive zones may exert a neuroprotective influence against PN glutamate excitotoxicity [17]. More widely, EAAT4 influences extrasynaptic transmission from PFs to adjacent Bergmann glia [18] and controls metabotropic glutamate type 1 receptor-mediated (mGluR1) synaptic events at PF and CF synapses to influence plasticity [19–21]. Without EAAT4, glutamate released by CF activation can even escape far enough to recruit basket cells and drive intersynaptic GABAergic transmission [22]. EAAT3 (EAAC1) is less abundant in the cerebellum, but like EAAT4, it locates to the somatodendritic compartment of the PN [12]. Interestingly EAAT3 (also known as EAAC1) is negatively modulated by its interacting protein, glutamate transporter-associated protein 3-18 (GTRAP3-18) [23] and is known to transport cysteine into cells to produce neuroprotective glutathione [24, 25].

The cerebellar cortex also richly expresses the astrocytic forms of the glutamate transporters, particularly EAAT1 (GLAST) and EAAT2 (GLT-1). Of the two, EAAT1 is most enriched in the Bergmann glial cells [26] and like EAAT4 also resides in close proximity to the PN somatodendritic compartment [1]. Uptake of glutamate by EAAT1 limits cross talk across cerebellar synapses; it controls the amplitude and time course of both CF-and PF-evoked AMPA receptor-mediated synaptic transmission [27] by preventing glutamate escape. EAAT1 mediated glutamate uptake also maintains the one to one innervation of the PN by the CF [28] and the independent operation of PF synapses [29]. Furthermore, during CF activation EAAT1 controls the spread of glutamate to nearby post-synaptic NMDA receptors on the PN spine [30] and also to presynaptic NMDA receptors on GABAergic terminals thereby also influencing local synaptic inhibition [31].

Here we show how the non-transportable blocker of EAATs 1-5, TBOA, (DL-threo-beta-benzyloxyaspartate, hereafter referred to as TBOA) adversely influences the behaviour of the PN and the PF-PN synapse, and discuss how these mechanisms might influence cerebellar synapse behaviour in the early stages of cerebellar ataxias. We used brief bursts of high frequency physiologically relevant PF activity to activate the PN (200 Hz EPSC), and show how TBOA enhanced the amplitude and duration of the PN EPSC in an mGluR1 dependent and independent manner. This enhanced depolarisation prolonged burst firing of the PN in response to PF input, even when mGluR1 mediated excitation was blocked. The excess glutamate made available by TBOA also recruited abnormal PF presynaptic mGluR4 signalling that modified the behaviour of the synapse. We predict that these mechanisms have the potential to contribute to mistimed cerebellar output during the onset and progression of some human cerebellar ataxias. Our findings also highlight the importance of developing ways to sustain cerebellar glutamate uptake as a useful early therapy to prevent the progression of cerebellar ataxia.

## Results

### Inhibition of glutamate transport enhanced high frequency stimulus-evoked parallel fibre EPSCs and revealed a slow EPSC with mGluR1 dependent and independent components

**Figure 1:**
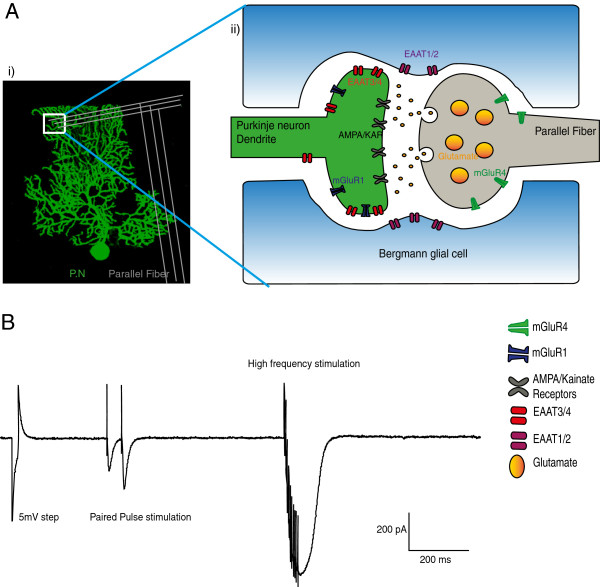
**Location of all the major transporters and receptors at a PF-PN synapse.** **(A)**(i) A biocytin filled Purkinje neuron post-hoc stained with streptavidin Alexa 488. (ii) A representative image of a Parallel fibre Purkinje neurons synapse showing the approximate locations of the major transporters and receptors targeted in this study. Parallel fibre synapses are located on the outer dendrites of Purkinje neurons, highlighted by the white box in (Ai). **(B)** An example trace showing our stimulation and recording protocol. We used a 5 mV step to calculate series and input resistance, followed by application of a pair of closely spaced stimuli to the parallel fibres and a high frequency burst stimulation (10 stimuli at 200 Hz), a protocol we repeated every 30 seconds.

**Figure 2:**
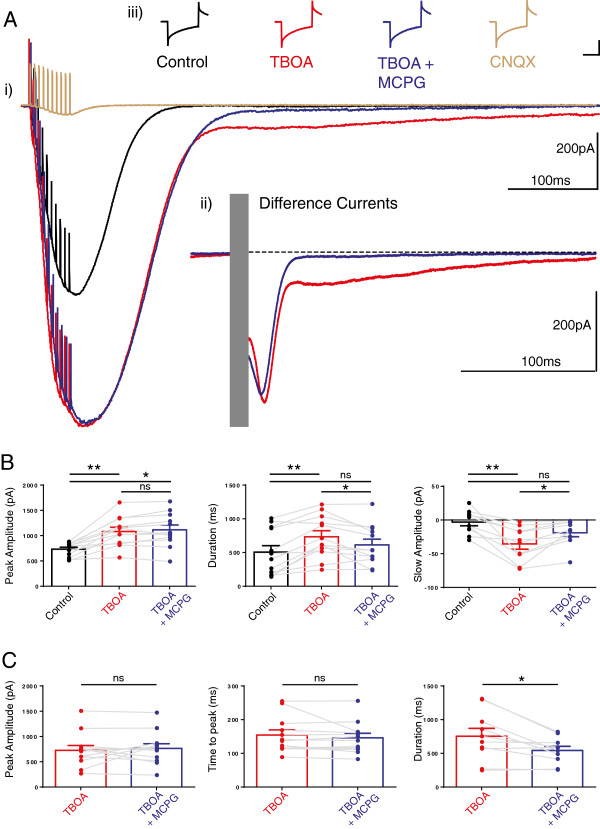
**Inhibition of glutamate transporters enhances PN responses to a short high frequency burst of parallel fibre activity.** **(Ai)** A burst of high frequency stimuli to the PFs evoked a large inward current in PNs (high-frequency PF EPSC). 50µM TBOA enhanced both the peak amplitude and the duration of this current, further addition of 0.2 mM MCPG, a type 1 mGluR antagonist significantly reduced the slow component of the high frequency EPSC leaving the peak current unchanged; CNQX, an AMPA-KA receptor antagonist abolished all current. Difference currents (Aii) where the inward currents before and after TBOA, and TBOA and MCPG, are subtracted. Note that in the presence of both TBOA and MCPG a small, slow current (note dashed line) remained, and this current was sensitive to the AMPA receptor antagonist CNQX (Ai). All changes occurred in the absence of any significant changes in input or series resistance of the neuron, scale bar is 100 pA and 10 ms (Aiii). **B** shows the individual and mean values of the changes in EPSC peak amplitude, duration and amplitude of the slow component of the high-frequency PF EPSC, bars are mean values and error bars are SEM, filled circles show values from individual neurons and connecting grey lines show how the responses changed in individual neurons; control, TBOA, and TBOA and MCPG. (Repeated measures oneway ANOVA, where * and ** represent P < 0.05, P < 0.01 respectively, ns denotes not significant.) **C** shows the mean difference currents for the same cells as in B. The amplitude and time to peak of the TBOA induced difference currents were unchanged by MCPG (P = 0.53 and 0.28 respectively) but the duration of the current significantly shortened, statistical comparisons used paired T-test, * represents P < 0.05.

A burst of ten, high frequency (200 Hz) stimulations to the PFs aimed to mimic the *in vivo* behaviour of PFs [32] and evoked a large amplitude long-lasting EPSC in cerebellar PNs, Figure 1 and Figure 2A, hereafter called the high-frequency PF EPSC. To inhibit glutamate transport we used 50 μM TBOA. This concentration of TBOA significantly and reversibly enhanced both the peak amplitude and the duration of the high-frequency PF EPSC, Figure 2B. These changes occurred in the absence of any changes in the series resistance, input resistance or holding current of the PN (see Methods below). TBOA application also revealed a larger amplitude slow component to the high-frequency PF EPSC that was reduced by the broad spectrum mGluR1 antagonist, MCPG (0.2 mM), leaving a smaller amplitude, slow EPSC remaining, Figure 2B. In the presence of the AMPA and Kainate receptor (KA-R) antagonist CNQX (6-cyano-7-nitroquinoxaline-2,3-dione) and TBOA and MCPG (see also Table 1 for explanation),the fast peak amplitude of the high-frequency PF EPSC reduced from 980 ± 167 pA in control to 112 ± 36 pA, n = 4, P < 0.001, *t*-test, whilst the slower component EPSC reduced from 65 ± 16 pA to 2 ± 1.2 pA (see Figure 2A). This result indicated that the majority of the TBOA-enhanced high-frequency PF EPSC resulted from AMPA-R (α-Amino-3-hydroxy-5-methyl-4-iso xazolepropionic acid receptors) and KA-R activation. The NMDA (glutamate) receptor antagonist APV (n = 6) did not influence the amplitude or duration of the high-frequency PF EPSC in the presence of TBOA (mean values of the EPSC changed from 771 ± 66 pA for control to 1087 ± 88.3 pA in TBOA, P < 0.05, one way ANOVA, but remained unchanged at 1128 ± 124 pA in the presence of TBOA and APV, not significant in one way ANOVA multiple comparison). We also observed reversibility of the effects of 50 μM TBOA in 4 cells 15 minutes after wash back; mean values of the amplitude and duration of the 200 Hz EPSC changed from 487 ± 64 pA to 674 ± 67 pA in TBOA and back to 503 ± 65 pA (F_2,11_ = 14.7, P < 0.05, one way ANOVA) and 291 ± 47 ms to 615 ± 118 ms and back to 379 ± 55 ms respectively (F_2,11_ = 4.4, P < 0.05, one way ANOVA). The time constant of the recovery of the 2^nd^ EPSC was also reversible, mean values changed from 19.3 ± 1.7 ms to 30 ± 3.2 and back to 22.9 ± 2.5 ms (F_2,11_ = 4.6, P < 0.05, one way ANOVA).

**Table 1:** **Table of major transporters and receptors involved in this study**

Receptor/Transporter	Location	Antagonist
EAAT1	Bergmann Glia	TBOA
EAAT2	Bergmann Glia	TBOA
EAAT3	Purkinje neuron (PN)	TBOA
EAAT4	Perisynaptic on PN	TBOA
AMPA/Kainate	PN, postsynaptic density (PSD)	CNQX
mGluR1	PN, edge of PSD	(S)-MCPG
mGluR4	Parallel fiber presynapse	MPPG

A list of major transporters and receptors used in this study along with their associated antagonists and locations. DL-TBOA = DL-*threo*-β-Benzyloxyaspartic acid, CNQX = 6-cyano-7-nitroquinoxaline-2,3-dione, (S)-MCPG = (*S*)-α-Methyl-4-carboxyphenylglycine, MPPG = (*RS*)-α-Methyl-4-phosphonophenylglycine.

Subtraction of the high frequency PF EPSC signal from the same signal in the presence of TBOA allows calculation of a difference current, seen in Figure 2A. This provides a quantitative index of the influence of glutamate transporter activity on the EPSC, so that without a functioning transporter we observe a greater current consistent with greater availability of glutamate at the synapse. The difference current also readily distinguishes the two distinct phases of the TBOA-enhanced high-frequency PF EPSC as additional AMPA-R, KA-R and mGluR1 receptors are recruited as a consequence of the greater availability of glutamate. The initial phase, that influences the peak of the EPSC, peaks at around 150 ms post stimulation, while the slower phase peaks approximately 400 ms later. MCPG reduced the slow phase leaving a small but measurable slow current, Figure 2C, but did not significantly influence the early phase suggesting that this is mediated exclusively by AMPA and KA-Rs, and as shown in Figure 2A, further addition of AMPA-KA receptor antagonist, CNQX, blocked all current.

Our findings confirm that a high frequency burst of PF activity is sufficient to activate glutamate uptake by TBOA sensitive glutamate transporters, consistent with a large body of previous literature. The difference current implies that under our experimental conditions, uptake peaks approximately 100 ms after stimulation and continues for several hundred milliseconds thereafter. The consequence of inhibition of glutamate uptake is that PF activation encourages a significant and long lasting depolarisation of the PN via the recruitment of AMPA, KA and mGluR1 receptors.

### Inhibition of glutamate transport enhanced Purkinje neuron firing in response to high frequency PF input that was refractory to the mGluR1 antagonist MCPG

In order to determine the consequence of reduced glutamate transport for PN excitability we next assessed how TBOA influenced spontaneous and burst evoked action potential firing behaviour. Since the difference currents (Figure 2A) indicate that the largest influence of reduced transporter activity occurs approximately 100 ms after the last burst and that the slower component peaks around 400 ms later, we analysed action potential firing over these times and compared them with pre stimulation firing.

**Figure 3:**
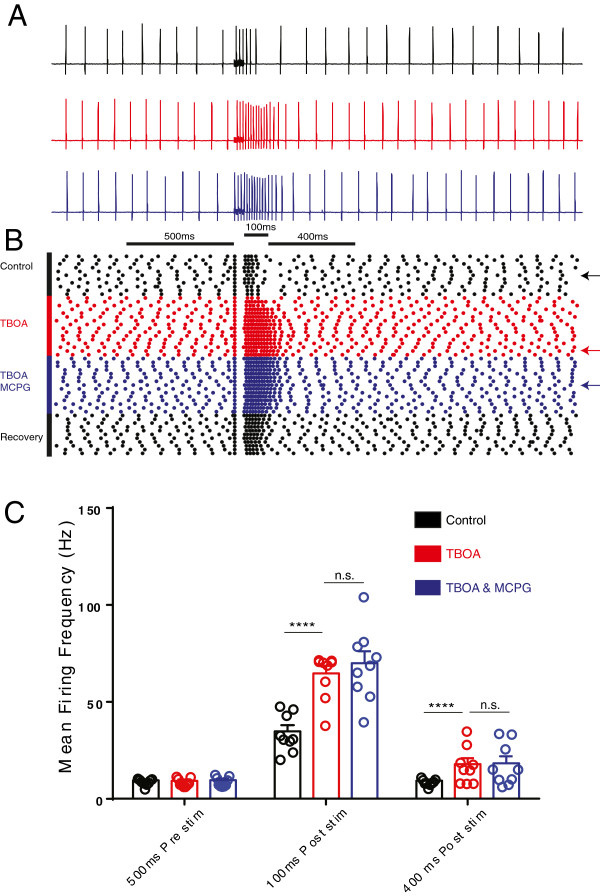
**Inhibition of glutamate transporters enhances immediate and prolonged burst firing of PNs in response to a short high frequency burst of parallel fibre activity.** **A** *,* example cell-attached recording from a PN reveals how action potential firing changes during the application of 50 μM TBOA and 50 μM TBOA in the presence of the mGluR antagonist 0.2 mM MCPG. Horizontal bar represents 200 Hz high frequency stimulation to PFs. **B**, shows a raster plot for the cell shown in *A,* each sweep (horizontal dots represent each action potential in the sweep) occurred at an interval of 30 seconds during the application of TBOA and TBOA and MCPG (left, vertical bars). Note the enhanced firing after the burst in the presence of TBOA and the inability of MCPG to reduce this. Arrows represent the raster plot for the traces shown above. **C**, bars show mean values (error bars are sem) of action potential firing frequency obtained from a 500 ms epoch just before stimulation, compared with action potential firing frequency at the peak of the TBOA-induced 200 Hz EPSC (during the 100 ms after the end of the stimulation) and later during the 400 ms after the stimulation when the slower, mGluR1 and EAAT4 dependent phase of the 200 Hz EPSC is active. **** represents P < 0.0001, two way ANOVA, ns is not significant.

As shown in Figure 3 TBOA significantly increased the firing frequency at both 0-100 ms and 100-500 ms after the stimulation, compared with basal, pre stimulation firing frequency (Row factor in two way ANOVA F_2,24_ = 96.8, P < 0.0001). Surprisingly, removal of mGluR1 mediated receptor activation (by MCPG) did not influence the post stimulation firing frequency (not significant in two way ANOVA multiple comparisons). TBOA also did not significantly influence the firing properties of the PN measured in the 500 ms before the PF burst, consistent with the lack of any significant change in holding current of individual PNs by 50 μM TBOA (see Methods).

### Inhibition of glutamate transport reduced paired pulse facilitation at the PF synapse via an mGluR4-mediated mechanism

Although reduced glutamate uptake clearly amplified PN excitability *per se* (Figure 2A) the excess glutamate under these conditions could also influence PF-PN synapse behaviour through presynaptic mechanisms. To test this we used the fact that the low release probability PF synapse exhibits the phenomenon of paired pulse facilitation (PPF). In this phenomenon, elevated residual presynaptic calcium encourages greater glutamate release in response to the second of a pair of closely spaced stimulations. Changes in presynaptic properties that reduce calcium influx are reliably predicted to reduce glutamate release and to enhance the facilitation of the second EPSC.

**Figure 4:**
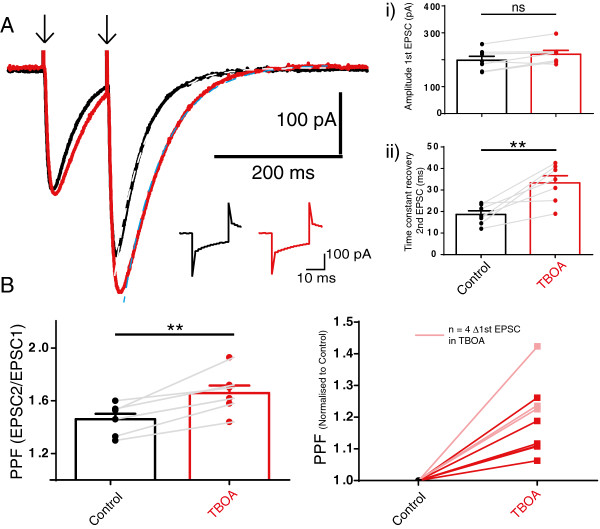
**Inhibition of glutamate transporters enhances paired pulse facilitation at the PF-PN synapse and prolongs the decay of the excitatory post synaptic current, EPSC.** **A**, shows an example recording from a PN showing EPSCs evoked with a pair of closely timed single stimulations to the PFs (downward arrows). Note the enhancement of the amplitude of the second EPSC upon application of 50 μM TBOA and the concomitant increase in duration of the second EPSC, in the absence of any change in the amplitude of the first EPSC (in 7/11 cells shown in *A(i)* where bars are mean ± SEM, ns is not significant in Student’s paired *t*-test). Dashed lines in *A* represent single exponential fits to estimate the time constant of recovery of the second EPSC; mean values (error bars are SEM) shown in *A(ii)* ** represents P < 0.01 Student’s paired *t*-test. Inset traces indicate no change in series resistance or input resistance by TBOA, see text, scale bar is 100 pA and 10 ms. **B** shows the change in mean absolute values of PPF in Control and after 50 μM TBOA, bars are mean ± SEM, ** represents P < 0.01 Student’s paired *t*-test. Left panel shows PPF change normalised to control for the 7/11 cells, bold colour, with remaining cells where the first EPSC increased, and PPF also increased, after TBOA, shown in a transparent shade.

As seen in Figure 4, TBOA increased PPF at the PF synapse. This result implies that excess glutamate availability reduced the probability of glutamate release at this synapse. Importantly, in 7/11 cells we observed this TBOA-enhanced PPF in the absence of any change in the amplitude of the first EPSC, as seen in Figure 4A. In the remaining 4/11 cells TBOA enhanced the 1^st^ EPSC and PPF, shown in Figure 4A, but we excluded these cells since interpretation becomes more complex under these circumstances. Note also that TBOA prolonged the recovery of the second of the pair of EPSCs, see Figure 4A, consistent with a slowed recovery of postsynaptic glutamate clearance.

In order to search for the mechanism underlying the TBOA-enhancement of PPF we omitted the high frequency stimulation that we routinely applied within our protocol (shown in Figure 1). Previous studies indicate that cannabinoid plasticity at this synapse only occurs in the presence of the high frequency bursts that mimic *in vivo* PF behaviour [33]. Under these conditions a brief burst of PF activity can cause mGluR1 activation and a localised increase in Ca^2+^ concentration. This subsequently leads to an endocannabinoid mediated suppression of glutamate release from the PF via the CB1 receptor [34, 35]. Similarly, when we omitted the high frequency stimulation TBOA failed to influence PPF (P = 0.16, n = 4, paired *t*-test). This strongly supported the idea that an activity-dependent plasticity mechanism contributed to the TBOA-induced enhancement of the PPF. However, we ruled out an mGluR1-mediated postsynaptic generation of a retrograde signal (such as from cannabinoid release from the PN, [33]) since TBOA continued to enhance PPF in the presence of 0.2 mM MCPG, the type 1 metabotropic glutamate receptor antagonist. Mean PPF was unchanged from 1.72 ± 0.07 to 1.73 ± 0.06, n = 12, not significant in multiple comparisons in a one-way ANOVA. Similarly, we excluded an action from presynaptic NMDA receptors at PFs [36] since the enhanced PPF persisted in the presence of TBOA + APV (an NMDA receptor antagonist). Mean values remained unchanged from 1.63 ± 0.04 to 1.64 ± 0.05, n = 6, not significant in multiple comparisons in a one-way ANOVA.

**Figure 5:**
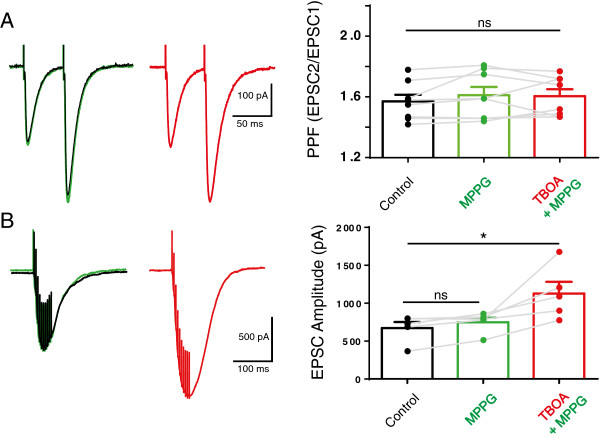
**The mGluR4 (type III) receptor antagonist MPPG specifically prevented the change in paired pulse facilitation (PPF) upon inhibition of glutamate uptake.** **A** shows that application of 50 μM TBOA in the presence of the mGluR4 antagonist MPPG no longer changed the PPF and that PPF was also not significantly changed by 25 μM MPPG alone. The graph shows the individual and mean values of the changes in PPF, bars are mean values and error bars are SEM, filled circles show values from individual neurons and connecting grey lines show response changes in individual neurons. The statistical comparison used a repeated measures oneway ANOVA where ns denotes not significant, F_2,23_ = 1.1, P = 0.35. Importantly the first EPSC was also unchanged (data not shown) in these experiments, F_2,23_ = 4.4, P = 0.08. TBOA still increased the time course of the recovery of the second of the pair of EPSCs, consistent with its postsynaptic action independent of the presence of MPPG, mean values changed from 14.7 ± 1.6 ms in control cells, to 14.2 ± 1.5 ms in MPPG alone treated cells to 21.7 ± 3.4 ms in MPPG and TBOA treated cells (F_2,23_ = 11.8, P < 0.05 repeated measures one way ANOVA). **B** shows for the same example cell as in A that the amplitude of the 200 Hz EPSC also increased in the presence of TBOA and MPPG, the graph is as for A above with a repeated measures oneway ANOVA where F_2,14_ = 8.7, P < 0.05 and multiple comparisons where * represents P < 0.05 and ns denotes not significant.

We therefore turned to mGluR4, a metabotropic glutamate receptor known to be expressed presynaptically at PFs [37] and known to depress their glutamate release probability [38]. As seen in Figure 5A, 25 μM MPPG, an antagonist of mGluR4 at PFs [39] abolished the TBOA-enhanced PPF. MPPG did not influence the TBOA-induced slower recovery of the second of the pair of EPSCs nor did it prevent the enhanced amplitude of the 200 Hz evoked large EPSC (both P > 0.05 in multiple comparisons from one way ANOVAs), see also Figure 5B. This strongly supports the action of excess glutamate at PF presynaptic mGluR4 receptors. MPPG also did not itself influence PPF, see Figure 5A, or the amplitude of the first EPSC (mean values were unchanged from control 204 ± 26 pA to 217 ± 32 in MPPG and 239 ± 39 pA in TBOA and MPPG, not significant in a one way ANOVA). Although KA-R also contribute to enhanced facilitation following high frequency PF stimulation [40] we did not observe any remaining PPF enhancement in the presence of TBOA and MPPG suggesting that presynaptic KA-R were unlikely to be recruited under our conditions.

## Discussion

Our findings illustrate that even a short period of reduced glutamate uptake adversely amplified synaptic responses at the PF-PN synapse to an extent that was sufficient to prolong PN firing output. Furthermore, reduced glutamate uptake also changed the timing and behaviour of the PF-PN synapse by the action of excess glutamate at presynaptic mGluR4 receptors.

The amplification of the PF-EPSC by TBOA is consistent with previous literature showing the importance of glutamate uptake at this and the climbing fibre (CF)-PN synapse [14–18]. The difference currents in Figure 2 show that glutamate uptake amplified both the fast and slow components of the PF response. The fast component likely represents excess glutamate acting at AMPA/KA receptors since it was abolished by CNQX. Likewise, the slow component was abolished by the type 1 metabotropic glutamate receptor antagonist MCPG, as shown by others at both PF and CF synapses [14, 19, 20], however a significant slow inward current remained. The fact that the AMPA-R antagonist CNQX abolished this slow MCPG-insensitive inward current in the PN indicates that it is a consequence of glutamate acting at glutamate receptors (including low affinity KA-R) [27]. Since previous work indicates that it is also absent in EAAT4 knockout mice [41] we propose that the slow current is driven by the opening of glutamate receptors (probably K-AR) as a consequence of excess available glutamate following EAAT4 inhibition by TBOA. The small, slow nature of the current is consistent with EAAT4 actions since EAAT4 is a high affinity glutamate transporter normally responsible for clearing the last remaining glutamate after PF stimulation; hence this slow current is most apparent in the presence of TBOA. In accordance with this previous literature we also observed a significant overlap between the mGluR insensitive, CNQX sensitive slow current (EAAT4 action) and the mGluR1-sensitive, CNQX insensitive current (mGluR1 action), Figure 2A. The overlap of timing of these two components is consistent with the idea that mGluR1 responses are limited in PNs within zebrin II-positive zones [20] where EAAT4 expression is highest [13] but are facilitated in PNs from EAAT4 knockout mice [18].

EAAT4 is known to have a large anion conductance compared to other EAATs [42], these anion currents, under resting conditions may inhibit cell excitability. It has also been speculated that EAAT4s hyperpolarising action could counteract the depolarising action of other EAATs, namely EAAT3. However, another report by Melzer [43] showed that during large depolarisations the EAAT4 transporter may become cation permeable and cause an excitatory inward current. A further consideration is that whilst EAAT4 currents may directly influence the local membrane potential in the PN dendrites they are unlikely to be directly responsible for the slow inward current recorded from the PN soma seen here, especially considering its susceptibility to CNQX.

### Inhibition of glutamate uptake prolongs Purkinje neuron firing output in response to PF input

We expected the sizeable fast and slow excitatory inward currents evoked by high-frequency PF stimulation in the presence of TBOA to alter the excitability of the PN. As seen in Figure 3, TBOA dramatically influenced PN firing behaviour, particularly in the 100 ms period immediately after the burst stimulation when TBOA-enhanced depolarisation was at its maximum (see Figure 2). Although this short lasting burst firing behaviour was reminiscent of CF activation the responses remained graded and did not exhibit the all-or-none behaviour characteristic of CF activation. The enhanced PN firing also persisted during the time when the mGluR1 and EAAT4 dependent current were active (Figure 3). It was therefore surprising that blockade of mGluR1 did little to influence PN firing (Figure 3C). This suggests that the small remaining slow CNQX sensitive current (presumed to be driven by EAAT4) is sufficient to drive the membrane potential above the PN action potential threshold. It is possible, since EAAT4 is uniquely expressed in the PN dendrites [12, 13] that this glutamatergic current exerts a far more powerful contribution in the dendrite and only appears small and slow from our recordings at the soma. Alternatively, or in addition, since TBOA blocks the EAAT4 chloride conductance this may directly enhance PN dendrite excitability to sustain bursting behaviour.

### Inhibition of glutamate uptake recruits PF presynaptic metabotropic glutamate receptors (mGluR4) to reduce glutamate release from PFs

Blockade of glutamate transporters also enhanced the phenomenon of paired pulse facilitation, a form of short term plasticity displayed by the PF synapse [44]. At this synapse, enhanced PPF indicates reduced presynaptic calcium and reduced glutamate release. We propose that TBOA reduced glutamate release may indicate an in-built plasticity mechanism to conserve overall synaptic weight at the PFs under conditions of excess glutamate [45, 46]. Indeed, depression of transporter current residual CF EPSCs in double EAAT4 EAAT1 knockout mice also suggests that excess glutamate reduces glutamate release at CF synapses [41]. Critically, the reduced glutamate release at PFs required inhibition of glutamate uptake AND high frequency bursts, indicative of an activity dependent mechanism. Of the possible candidates, neither NMDA nor mGluR1-mediated mechanisms influenced the TBOA-induced change in PPF, rather it was prevented by the mGluR4 antagonist MPPG, a finding consistent with the unique presence of mGluR4 at PFs [37, 38]. These receptors reduce glutamate release from PFs by reducing calcium influx [47] and by directly interacting with the release machinery [48, 49]. MPPG alone did not change the behaviour of the PF-PN synapse suggesting that the combination of excess glutamate (by TBOA) and high-frequency stimulation together enabled sufficient glutamate to reach the PF presynaptic mGluR4 receptors. Indeed, a previous study showed that several seconds of PF stimulation is needed to activate mGluR4 [50]. The important consequence of mGluR4 recruitment in the face of excess glutamate is to usefully weaken, or balance, the PF-PN synapse. It is tempting to speculate that a similar mechanism contributes to long term depression (LTD) at this synapse, a phenomenon traditionally linked to motor learning. Interestingly, an orally active mGluR4 positive allosteric modulator influences motor behaviour [51].

## Conclusions

### Relevance for cerebellar ataxia

Our findings predict that loss of glutamate transporter activity leads to excessive and mis-timed PN output and altered PF plasticity that could lead to defective cerebellar output. Such changes are likely to take place during pre-symptomatic or early stages of a variety of human ataxias and mouse models of human ataxias [2–7]. Our results imply that chronic loss of transporter activity during progression of ataxia will be accompanied by hyperexcitability, oxidative stress, excitotoxicity and death of PNs. Since PN death is the end point of the disease novel therapeutic approaches to sustain EAAT transporter activity in the early phases of the disease merit serious attention. Possible approaches include reduced expression of GTRAP3-18, a protein that normally reduces EAAT3 activity [23], activation of innate compensatory mechanisms to elevate glutamate uptake [52] or selective stimulation of JAK kinases known to elevate EAATs [53], all of which may hold promise as future treatment strategies.

## Methods

### Mice

We used male C57 B6 mice aged 22-30 days. All animal husbandry and ethical procedures minimized animal suffering and followed internationally recognized standards as part of University of Otago approved guidelines in accordance with New Zealand Animal Welfare Act (1999).

### Electrophysiology

300 μm thick saggital slices from the cerebellar vermis were prepared from mice following rapid anaesthesia with carbon dioxide. Slices were maintained at 24°C (TC324B, Harvard Apparatus, USA) in a flow (3 ml/min) of artificial cerebrospinal fluid (aCSF), equilibrated with 95% O_2_ and 5% CO_2_, containing (in mM) NaCl 126, KCl 2.5, NaH_2_PO_4_ 1.2, MgCl_2_ 1.3, CaCl_2_ 2.4, NaHCO_3_ 26, glucose 10. Whole cell recordings from PN soma under visual control used glass electrodes (3-5 MΩ) containing (in mM) KCl 4.5, KOH 20, MgCl_2_ 3.48, NaCl 4, K gluconate 120, Hepes 10, sucrose 8, Na_2_ATP 4, Na_3_GTP 0.4, 10 mM EGTA pH 7.3 and osmolarity 305 mOsm/l. Voltage clamp (Axopatch 200B, Molecular Devices, USA) maintained cells at -70 mV holding current for at least 5 minutes prior to electrophysiological protocols (PClamp, Molecular Devices, USA). Mean uncompensated series resistance was 18 ± 1.1 MΩ in control and 17.5 ± 1.2 MΩ in 50 μM TBOA, P > 0.05, n = 12, paired *t*-test; TBOA also did not influence PN input resistance or holding current, mean values were unchanged from 85.2 ± 3 to 87.6 ± 3.7 MΩ and from -154.7 ± 21.9 pA to -188.8 ± 29.7 pA, both P > 0.05, n = 12, paired *t*-test. PF stimulation (10 μs, 2-20 V, constant voltage stimulator, Digitimer, UK). Capacitance and input resistance were estimated from the current change during a 5 mV step. We used a pair of stimuli for PPF measurements separated by an inter-stimulus interval of 50 ms, and also a burst stimulus delivered 550 ms post PPF stimulation, consisting of 10 stimuli at 200 Hz (5 ms interval) (see Figure 1). Both types of stimulus were delivered at 0.011 Hz (every 30 seconds) using a glass aCSF-filled monopolar stimulation electrode (200-600 kΩ) placed in the outer two thirds of the molecular layer approximately 120-150 μm from the recorded cell and the burst stimulus followed the PPF stimulation Figure 1). Loose patch recordings (Axopatch 200B) from the soma of PNs used glass electrodes with a resistance of approximately 6 MΩ employing the same internal solution as above.

### Solutions and pharmacological treatments

EPSCs were evoked in the presence of 50 μM picrotoxin (Sigma-Aldrich, UK) and prevented by 20 μM CNQX, (1,2,3,4-Tetrahydro-6-nitro-2,3-dioxo-benzo[f]quinoxaline-7-sulfonamide (Tocris Cookson). Electrophysiology protocols were repeated after 10-15 minutes bath application of TBOA (50 μM, (Sigma-Aldrich) a broad spectrum inhibitor of glutamate transport (10-50 μM, Tocris Cookson). Pharmacological tools included 0.2 mM (S-)MCPG, (S)-α-Methyl-4-carboxyphenylglycine and 25 μM MPPG, (RS)-α-Methyl-4-phosphonophenylglycine all stored as a 1000× stock solution in DMSO or water. (See Table 1 for list of antagonists used).

Control applications of vehicle instead of TBOA over the same time period revealed no significant changes in any of our measured parameters (P > 0.05, n = 4, paired t-tests).

### Analysis and statistics

Offline analysis of all electrophysiological data used PClamp 9 (Molecular Devices, USA). Slow inward current amplitudes were measured at 500 ms after the onset of the high frequency stimulation, time constants of recovery of EPSCs used a single exponential fit. Statistical analysis was performed in Prism 6.0 (GraphPad) and Student’s *t*-test and one and two way ANOVAs as appropriate.

## Abbreviations

aCSF: Artificial cerebrospinal fluid
AMPA-R: Alpha-Amino-3-hydroxy-5-methyl-4-isoxazolepropionic acid receptors
CF: Climbing fibres
CNQX: 6-cyano-7-nitroquinoxaline-2,3-dione
EAAT: Excitatory amino acid transporter
GTRAP: Glutamate transporter associated protein
KA-R: Kainate receptors
MCPG: (S)-α-Methyl-4-carboxyphenylglycine
mGluR: Metabotropic glutamate receptor
MPPG: (RS)-α-Methyl-4-phosphonophenylglycine
PF: Parallel fibre
PN: Purkinje neuron
PPF: Paired pulse facilitation
SCA: Spinocerebellar ataxia
TBOA: DL-threo-beta-Benzyloxyaspartic acid.
